# Developing and validating multi-scenario EFL willingness to communicate scale across classroom, extracurricular, and GenAI-mediated scenarios

**DOI:** 10.1186/s40359-026-04607-1

**Published:** 2026-04-22

**Authors:** Weihe Zhong, Zhiyi Zhou, Lifei Wang, Yanchao Yang

**Affiliations:** 1 Rector’s Office, Macau Millennium College, 8th Floor of China Civil Plaza, No. 255, Alameda Dr. Carlos D’Assumpção, Macau SAR, 999078 People’s Republic of China; 2 School of English and International Studies, Beijing Language and Culture University, No.15 Xueyuan Road, Haidian District, Beijing, 100083 P.R. China; 3 Institute of International Language Services Studies, Macau Millennium College, 8th Floor of China Civil Plaza, No. 255, Alameda Dr. Carlos D’Assumpção, Macau SAR, People’s Republic of China

**Keywords:** EFL, WTC, GenAI, Classroom, Extracurricular, Scale Development, Scale Validation

## Abstract

With the widespread application of generative artificial intelligence (GenAI) in language learning, interaction with GenAI has become an important form of English-speaking practice. However, existing Willingness to Communicate (WTC) in EFL context scales have not yet adequately captured this emerging context. Therefore, this study aims to extend the theoretical framework of English WTC in the era of GenAI and to develop and validate a multi-scenario WTC scale that encompasses classroom, extracurricular, and GenAI-mediated scenarios. Using a sample of 1,899 university students, the study employed principal component analysis, confirmatory factor analysis, correlation analysis, and multi-group invariance testing to systematically examine the reliability and validity of the scale. The results indicate that the scale demonstrates good reliability and validity, and exhibits strong measurement invariance across gender and household registration groups.

**Supplementary Information:**

The online version contains supplementary material available at 10.1186/s40359-026-04607-1.

## Background

Artificial intelligence—especially the rapid development of GenAI—is profoundly transforming traditional language learning models [1–3]. Artificial intelligence is driving transformative changes across all dimensions of language learning—from listening comprehension [4] and reading comprehension [5] to spoken expression [6] and writing proficiency [7]. In the domain of English speaking practice, GenAI chatbots are leveraging advanced natural language generation and intelligent conversational capabilities to create a low-pressure, highly immersive environment for learners. Recent years have seen remarkable advances in AI-powered speech training technologies, including accurate speech recognition [8], intelligent sentiment and emotion analysis [9], fluent and natural-sounding speech synthesis [10], and personalized feedback mechanisms [11]. Together, these innovations are fundamentally reshaping how language learners engage with and develop their oral communication skills.

This shift towards AI-mediated speaking practice can be directly linked to the concept of WTC. By providing a non-judgmental and supportive environment, these AI systems lower anxiety and offer tailored feedback, thereby enhancing learners’ WTC and encouraging greater oral communication in English [12–14]. In the context of English speaking development, learners’ WTC is a critical factor [15]. Traditional research has primarily focused on WTC in real-life interpersonal settings—such as classroom interactions [16] or extracurricular contexts [17]. With the growing integration of artificial intelligence in education, human–AI interaction is increasingly emerging as a novel mode of language practice. Such interactions can be characterized by quasi-interpersonal interaction characteristics, whereby AI systems simulate human-like conversational exchanges through context-aware dialogue, adaptive feedback, and responsive interaction patterns, making human–AI interaction an increasingly meaningful communicative context for language practice. Unfortunately, existing studies on WTC scales in English have yet to adequately address this evolving landscape, resulting in a notable gap: the absence of validated instruments capable of accurately measuring learners’ WTC in English within GenAI–mediated scenarios.

## Literature review

### Willingness to communicate

The concept of WTC originally emerged from research on native language communication, stemming from Burgoon’s [18] notion of Unwillingness to Communicate. This concept describes a chronic tendency to avoid or devalue verbal interactions, often rooted in communication anxiety, low self-esteem, fear, or feelings of alienation [18]. Building on this, WTC was later defined as an individual’s predisposition to initiate or engage in conversation under specific circumstances [19]. Traditionally, it was viewed as a stable personality trait unaffected by the environment. However, McCroskey and colleagues argued that WTC is actually a situational variable influenced by various factors.

While L1 WTC is predominantly conceptualized as a stable personality trait reflecting an individual’s general inclination to communicate, L2 (and specifically EFL) WTC represents a more complex construct: it is the willingness to use a specific language (English) as the medium of interaction. In the 1990s, MacIntyre and others introduced this concept into the field of second language acquisition, proposing the idea of L2 WTC [20, 21]. They highlighted its uniqueness within a second language context—specifically, the extent to which second language learners choose to use the target language for communication at a particular time, with certain people, or in specific situations. To explain the mechanisms underlying L2 WTC, MacIntyre and colleagues developed a heuristic pyramid model [20]. This model illustrates how multiple factors such as learners’ L2 motivation, confidence, and communicative competence interact to shape their WTC in a second language context, thereby influencing actual language use behaviors. It suggests that learners’ WTC significantly determines their real-world communicative actions.

The evolution of WTC from native language to second language research not only reflects an expansion of research domains but also reveals a profound shift in conceptual understanding. In native language studies, WTC was understood as a stable disposition determined by personality traits. However, upon entering the realm of second language acquisition, its essence transformed. L2 WTC became a dynamic decision-making process influenced by linguistic proficiency, psychological states, and social contexts, offering a new theoretical perspective for understanding silence phenomena in second language learning and designing instructional strategies to promote communication. This transformation underscores the importance of considering both internal and external influences on learners’ willingness to engage in second language communication.

Unlike learners in English as a Second Language (ESL) settings, who often have access to natural environments where English is spoken, learners in English as a Foreign Language (EFL) contexts, such as China and Japan, typically lack such opportunities. Consequently, EFL learners’ WTC relies more heavily on artificially created contexts like classroom discussions, role-plays, or simulated tasks [22], which are not driven by immediate needs, making the development of WTC more challenging. Beyond classrooms, school-organized English activities—such as English corners, competitions, or interactions with peers and native speakers—offer more authentic communication scenarios [17]. However, the impact of these activities remains limited by their frequency and participation levels, affecting both the frequency of English usage and learners’ initiative to engage in communication.

The rapid advancement of GenAI technologies has introduced new opportunities for learners to enhance language skills through interactions with AI tools like ChatGPT. Rather than functioning merely as technological tools, these AI-mediated environments can also be conceptualized as dialogic spaces where learners engage in ongoing interaction, negotiation of meaning, and responsive exchanges with intelligent systems. Such environments provide opportunities for learners to participate in simulated conversational dialogue, which may encourage active language use and foster greater willingness to communicate. According to Krashen’s Affective Filter Hypothesis [23] negative emotions such as anxiety, fear, and low self-confidence can create an “affective filter” that impedes language input absorption. AI systems, characterized by their high patience, non-judgmental nature, and ability to offer repeat interactions, can effectively lower learners’ anxiety levels and reduce this affective filter, indirectly boosting their WTC. Furthermore, in MacIntyre et al.‘s [20] situational model of L2 WTC — often visualized as a heuristic pyramid — willingness to communicate is conceptualized not only as a stable trait but also as a highly dynamic, situation-specific readiness to enter discourse, immediately preceded by state communicative confidence and the desire to communicate with a specific interlocutor. The “social presence” of advanced GenAI [24], enhanced by natural language processing and affective computing, uniquely interacts with this situational layer: unlike traditional learning tools, GenAI can simulate a consistent, responsive, and affectively attuned conversation partner, reflecting quasi-interpersonal interaction characteristics, thereby increasing the perceived affiliation and emotional mutuality with the interlocutor — even though it is non-human. This heightened sense of “being with” a safe yet realistic interactant directly strengthens learners’ momentary desire to engage and bolsters their state-level self-confidence in the here-and-now of interaction, going beyond anxiety reduction to actively shape the proximal antecedents of WTC in the situational model. Highly socially present AI systems can provide more natural language feedback and emotional responses during conversations, increasing the realism and immersion of interactions, thus encouraging learners to engage more willingly and authentically.

From a social cognitive theory perspective, individual learning and behavioral development result from dynamic interactions between environmental factors, cognitive factors, and behaviors. AI-based virtual communication scenarios and intelligent dialogue tools serve as critical environmental factors, offering precise, instant linguistic feedback [12, 25] This allows learners to iteratively refine their language output, observe improvements, and gradually build self-efficacy. Enhanced self-efficacy fosters positive perceptions of one’s communicative competence, sustaining motivation for continuous practice and expression even in the absence of real communicative contexts. This, in turn, boosts learners’ confidence and WTC.

In the Chinese context, where cultural factors such as ‘face’ (mianzi) play a pivotal role, EFL learners often experience heightened anxiety about making errors and facing peer judgment. This anxiety significantly inhibits their willingness to communicate, particularly when they are required to perform tasks in class or participate in extracurricular speech contests. In stark contrast, GenAI-mediated scenarios offer a private, non-judgmental space where the fear of ‘losing face’ is virtually eliminated [12]. The absence of human evaluation allows learners to experiment with English without social risk, thereby uniquely boosting their WTC in this specific context [14]. These divergent behavioral patterns across public classroom settings, extracurricular activities, and private GenAI interactions underscore a critical theoretical insight: WTC in EFL is not a monolithic trait but a highly situational and multi-contextual phenomenon. Consequently, a generic scale fails to capture these nuances, necessitating the development of a specialized instrument that explicitly differentiates between these distinct communicative ecologies. This need for a context-sensitive measurement approach forms the core contribution of the present study.

However, existing WTC scales primarily focus on learners’ willingness to communicate in traditional interpersonal contexts, such as classroom or extracurricular interactions. Even when digital environments are considered, communication typically remains human-to-human, with technologies such as social media or messaging platforms merely functioning as communication channels. In such contexts, learners are still subject to social evaluation, peer judgment, and interpersonal dynamics that may heighten anxiety or constrain spontaneous language use. Consequently, current scales insufficiently capture learners’ willingness to communicate in emerging interaction contexts, particularly human–AI communication scenarios enabled by generative AI technologies. Unlike digital human-to-human interaction, GenAI-mediated communication is characterized by quasi-interpersonal features, including non-judgmental feedback, adaptive personalization, and the ability to sustain low-pressure, repeatable interactions. These features fundamentally alter learners’ affective experiences and perceived communicative risks, creating a distinct communicative ecology. Addressing this gap can provide a more comprehensive understanding of how learners’ willingness to communicate evolves across different contexts, especially in increasingly prevalent AI-enhanced learning environments.

### Measuring willingness to communicate in English

Existing research on EFL learners’ WTC in English has primarily focused on measuring communicative intent within interpersonal, face-to-face contexts. One of the earliest and most influential instruments is the WTC scale developed by McCroskey and Baer [19]. Originally designed for native-language communication, this psychological tool assesses individuals’ propensity to engage in verbal interaction across three types of interlocutors (strangers, acquaintances, and friends) and four communication settings (public speaking, meetings, group discussions, and dyadic conversations). However, as the scale was validated mainly in bilingual or L1 contexts, its applicability to second language learning environments remains limited [26].

Building on this foundation, MacIntyre et al. [22] developed a scale specifically tailored to L2 contexts, measuring WTC across four language skills—speaking, listening, reading, and writing—in both classroom and extracurricular situations. Nevertheless, Weaver [27] noted that some items in this scale were heavily influenced by native-language norms, and the scenarios described often do not reflect common activities in typical language classrooms, thereby constraining its validity [26].

Meanwhile, Ryan [28] introduced an eight-item scale using a 6-point Likert format to assess WTC in English or Japanese across various social contexts. The items describe concrete situations such as giving a speech in front of an audience, chatting with strangers or acquaintances while waiting in line, interacting with sales staff, and participating in group discussions. This design enables cross-linguistic and cross-contextual comparisons of WTC. More recent studies have further contextualized WTC measurement. For instance, Peng and Woodrow [16] focused on classroom-specific behaviors, such as using English in role-plays or expressing ideas to peers. Similarly, Lilya [17] developed a scale that examines learners’ tendencies to use English when communicating with native speakers, distinguishing between real-life and online environments.

In summary, although existing research has developed various scales to assess learners’ WTC across classroom, extracurricular, skill-specific, and everyday contexts, these instruments largely overlook digital and virtual communicative environments. In particular, they fail to account for emerging human–AI interaction scenarios, which differ structurally and psychologically from traditional face-to-face communication. As a result, current measures do not fully capture learners’ willingness to communicate in increasingly technology-mediated settings. This limitation highlights the need for a more contextually inclusive instrument capable of assessing learners’ dynamic WTC across both traditional and GenAI-mediated communicative environments.

## Method

### Participants

A total of 1,899 university students from institutions in Hebei, Henan, Beijing, and Chongqing participated in this study. The sample was randomly divided into three groups using SPSS software. The first subsample (*n* = 633) was used for exploratory factor analysis, comprising 136 males and 497 females; 163 participants held urban household registration, and 470 held rural household registration. The second subsample (*n* = 633) was used for confirmatory factor analysis, including 130 males and 503 females; 164 with urban household registration and 469 with rural household registration. The third subsample (*n* = 633) was used for multi-group confirmatory factor analysis, consisting of 120 males and 513 females; 120 with urban household registration and 513 with rural household registration.

### Measures

#### Multi-scenario EFL willingness to communicate scale

The scale developed in this study aims to assess students’ WTC in a foreign language across different contexts. Its dimensions are based on real-life situations in which students use English and are categorized into three aspects: classroom scenario, extracurricular scenario, and GenAI-mediated scenarios. First, the research team designed a semi-structured interview protocol by drawing on existing scales [16, 17] and conducted focus-group interviews with four English teachers familiar with AIGC technologies. Focus-group interview lasted approximately 30 min. During the interviews, participants were invited to discuss situations in which learners may be willing to communicate in English across different contexts, including classroom activities, extracurricular interactions, and emerging GenAI-mediated environments. The discussions focused on identifying representative communication scenarios that could reflect learners’ willingness to use English in these contexts. Insights from these discussions were used to inform the development of the initial item pool for the scale. The interview transcripts were subjected to a thorough qualitative analysis using a thematic approach. The research team conducted open coding on the transcripts, systematically identifying and categorizing key behaviors related to students’ WTC in English in the classroom, extracurricular activities, and GenAI-mediated scenarios. During the item selection process, the three dimensions were refined based on the contextual characteristics of different communication settings. Items retained for the classroom dimension primarily reflected typical instructional activities, such as answering teachers’ questions, reading aloud, and engaging in task-based interactions (e.g., role-playing), which represent structured and teacher-guided communication. Items for the extracurricular dimension were selected to capture common out-of-class English use, including participation in competitions, clubs, and informal communication with peers or other English users, reflecting more spontaneous and socially embedded interaction contexts. In contrast, items for the GenAI-mediated dimension were specifically selected to represent how learners use AI technologies for language practice. Rather than including general or repetitive interaction forms, priority was given to items that reflect function-oriented uses of GenAI, such as simulating daily conversations or interview scenarios, engaging in topic-based discussions, seeking targeted language support (e.g., pronunciation improvement), and practicing formal or academic communication. This selection process ensured that the GenAI items capture distinctive human–AI interaction patterns, thereby differentiating them from both classroom-based and human-to-human digital communication contexts. During the iterative integration phase, the team carefully considered whether to combine, eliminate, or retain specific items. Some items that were too similar in meaning were merged to improve clarity and reduce redundancy. For instance, items that focused on similar types of activities, such as “exploring social issues, technological developments, etc.,” were consolidated as examples of topics for discussion or debate with GenAI tools. This consolidation ensured that the items effectively captured distinct behaviors without unnecessary overlap, making the scale more concise and focused. Several items were retained with slight modifications by adding examples to ensure clarity and precision. To ensure the accuracy and relevance of the items, a member checking process was conducted. After the initial coding and integration, the team revisited the items and cross-checked them with the original interview data to verify that the themes and behaviors represented in the items were aligned with the participants’ responses. As a result, an initial pool of 17 items was developed, reflecting students’ WTC in English across the three scenarios: classroom, extracurricular, and GenAI-mediated scenarios (see Appendix 1). Subsequently, eight experts in English language teaching evaluated all items for content validity using a four-point relevance scale (1 = not relevant, 2 = somewhat relevant, 3 = quite relevant, 4 = highly relevant). All items received ratings of 4 from all experts, indicating unanimous agreement regarding their relevance. Consequently, the Item-Level Content Validity Index (I-CVI) for each item was 1.00, and the Scale-Level Content Validity Index (S-CVI) was also 1.00, demonstrating excellent content validity. Therefore, all 17 items were retained for subsequent analyses. The final scale employs a 5-point Likert rating format, ranging from “Strongly Disagree” (1) to “Strongly Agree” (5), with higher total scores indicating greater WTC in English as a foreign language.

#### Scale of willingness to communicate in English

This study adopted the Scale of Willingness to Communicate in English developed by Peng et al. [16] as the criterion instrument. The scale was adapted from Weaver’s [27] questionnaire. It uses a five-point Likert scale and consists of two dimensions: WTC in English Meaning-focused Activities (WTCMFACT), which includes six items (e.g., “I am willing to participate in group discussions in English”), and WTC in form-focused activities (WTCFFACT), which includes four items (e.g., “I am willing to answer grammar exercise questions in English”). This scale has been widely used in second language acquisition research both in China and internationally and demonstrates stable psychometric properties. In the present study, the Cronbach’s α coefficient for the meaning-oriented dimension was 0.958, and that for the form-oriented dimension was 0.961, indicating that the scale showed good reliability in the current sample.

### Analytical procedure

This study followed a systematic scale development and validation protocol to examine the reliability, validity, and structural robustness of the Multi-scenario EFL WTC Scale. To evaluate item discriminability, an item analysis was first conducted with SPSS 28 using extreme group (high–low) comparisons to assess each item’s ability to differentiate participants based on total scores. Additionally, item-total correlations and Cronbach’s α coefficients were computed to evaluate internal consistency at both the subscale and overall scale levels. Furthermore, “Cronbach’s alpha if item deleted” was computed for each item to determine whether removing any item would improve the scale’s reliability, thereby informing potential item deletion decisions.

Principal component analysis (PCA) was employed with SPSS 28 to determine the scale’s factor structure. Under the condition that the Kaiser–Meyer–Olkin (KMO) measure and Bartlett’s test of sphericity indicated suitability for factor analysis, the number of factors was predetermined based on theoretical considerations, and factors were extracted using varimax rotation. Items were retained if they met the following criteria: (1) factor loading ≥ 0.40 (2), cross-loading ≤ 0.30, and (3) conceptual coherence with the intended construct.

Subsequently, confirmatory factor analysis (CFA) was performed with the AMOS 28 on the second subsample to evaluate model fit using the following indices: χ²/df < 3, Comparative Fit Index (CFI) ≥ 0.90, Tucker–Lewis Index (TLI) ≥ 0.90, Root Mean Square Error of Approximation (RMSEA) ≤ 0.08, and Standardized Root Mean Square Residual (SRMR) ≤ 0.05 [29, 30]. However, the chi-square test is sensitive to sample size, and with large samples, even small deviations may lead to a significant chi-square value [31]. Therefore, relying solely on the chi-square test to assess model fit can be problematic due to its sensitivity to sample size. To address this issue, this study chose a χ²/df of less than 5 as the criterion for model fit to reduce the bias introduced by sample size [32]. In addition to this, other fit indices were also considered to provide a more comprehensive assessment of the model fit. These indices, including CFI, TLI, RMSEA, and SRMR, helped ensure a more robust evaluation of model fit, mitigating the limitations of the chi-square test alone. Convergent validity was assessed using the following benchmarks: standardized factor loadings > 0.70, Average Variance Extracted (AVE) ≥ 0.50, and Composite Reliability (CR) ≥ 0.70. Discriminant validity was evaluated using the Fornell–Larcker criterion: a latent construct demonstrates adequate discriminant validity if the square root of its AVE exceeds its correlations with all other latent constructs. Criterion-related validity was examined using SPSS 28 by correlating Multi-scenario EFL WTC Scale with Peng et al.’s scale [16]—a validated instrument adapted from Weaver’s scale [27]—used as the criterion measure.

Finally, to test measurement invariance across subgroups (e.g., gender, household registration type), multi-group confirmatory factor analysis (MG-CFA) was conducted with AMOS 28 using the third subsample. Configural, metric, scalar, and residual invariance were tested sequentially. Because the χ² difference test is highly sensitive to sample size and may indicate significant differences even when the practical change in model fit is minimal, changes in model fit between nested models were evaluated using |ΔCFI| < 0.01, |ΔTLI| < 0.01 [33], and |ΔRMSEA| < 0.015 [34] as thresholds for acceptable invariance.

### Research ethics

This study received ethical approval from the institutional review board of the affiliated institution (Approval No. MMCIRB-2024-002). An informed consent form was embedded in the online questionnaire, and participants were required to click “Agree” before proceeding, ensuring voluntary participation. No personally identifiable information was collected during the research process, and all data were used solely for academic research purposes, with anonymity and confidentiality fully ensured.

## Results

### Item analysis

To evaluate the discriminative power of the items, item analysis was conducted using the first subsample. The high–low group method (the 27% rule) was employed to assess item discrimination. Participants with total scores of 56 or below were classified into the low-score group, while those with total scores of 68 or above were classified into the high-score group, and independent-samples t tests were then performed. The results showed that all items exhibited significant differences between the high- and low-score groups (*p* < 0.05). Furthermore, to examine item consistency, item–total correlation analysis was conducted. The results indicated strong correlations between each item and the total scale score, ranging from 0.727 to 0.864. In addition, analysis of Cronbach’s α coefficient showed that the scale demonstrated extremely high internal consistency (Cronbach’s α = 0.969). Moreover, the α value did not change substantially when any single item was deleted, indicating a high degree of consistency between the individual items and the overall scale structure. Therefore, all items were retained.

### Principal component analysis

To determine the factor structure of the scale, Kaiser–Meyer–Olkin (KMO) and Bartlett’s test of sphericity were first conducted to examine whether the data were suitable for principal component analysis. The results showed a KMO value of 0.958, and Bartlett’s test of sphericity was significant (χ² = 13,372.455, df = 136, *p* < 0.001), indicating that the data were appropriate for principal component analysis.

Based on theoretical assumptions, a fixed three-component extraction method was adopted, and varimax rotation was applied to enhance the interpretability of the factors. After five iterations, the solution converged, and three principal components were ultimately extracted as shown in Table 1. These components corresponded to WTC in foreign languages in GenAI–mediated scenarios, classroom contexts, and extracurricular contexts, respectively, consistent with the hypothesized scenarios.

**Table 1:** Rotated Component Matrix

Item	Component 1	Component 2	Component 3	Communality	Variance explained	Cronbach’s α
GenAI1	0.215	**0.873**	0.213	0.854	27.700%	0.962
GenAI2	0.219	**0.857**	0.285	0.863		
GenAI3	0.232	**0.875**	0.253	0.883		
GenAI4	0.258	**0.873**	0.220	0.877		
GenAI5	0.229	**0.873**	0.223	0.865		
Ex1	0.399	0.249	**0.782**	0.832	27.328%	0.955
Ex2	0.406	0.254	**0.790**	0.852		
Ex3	0.387	0.386	**0.705**	0.796		
Ex4	0.404	0.295	**0.776**	0.853		
Ex5	0.449	0.231	**0.755**	0.825		
Ex6	0.395	0.310	**0.718**	0.767		
Cr1	**0.799**	0.271	0.407	0.877	29.158%	0.961
Cr2	**0.776**	0.257	0.437	0.858		
Cr3	**0.792**	0.313	0.332	0.835		
Cr4	**0.792**	0.256	0.377	0.835		
Cr5	**0.814**	0.254	0.365	0.859		
Cr6	**0.735**	0.210	0.442	0.780		

*Extraction method Principal* Component Analysis. *Rotation method* Kaiser-normalized Varimax rotation. Rotation converged after 5 iterations. *p*=0.01

Specifically, the classroom scenario component explained 29.158% of the variance, with a Cronbach’s α coefficient of 0.961; the extracurricular scenario component explained 27.328% of the variance, with a Cronbach’s α coefficient of 0.955; and the GenAI-mediated scenario component explained 27.700% of the variance, with a Cronbach’s α coefficient of 0.962. Together, the three components accounted for 84.185% of the total variance.

### Confirmatory factor analysis

**Fig. 1:**
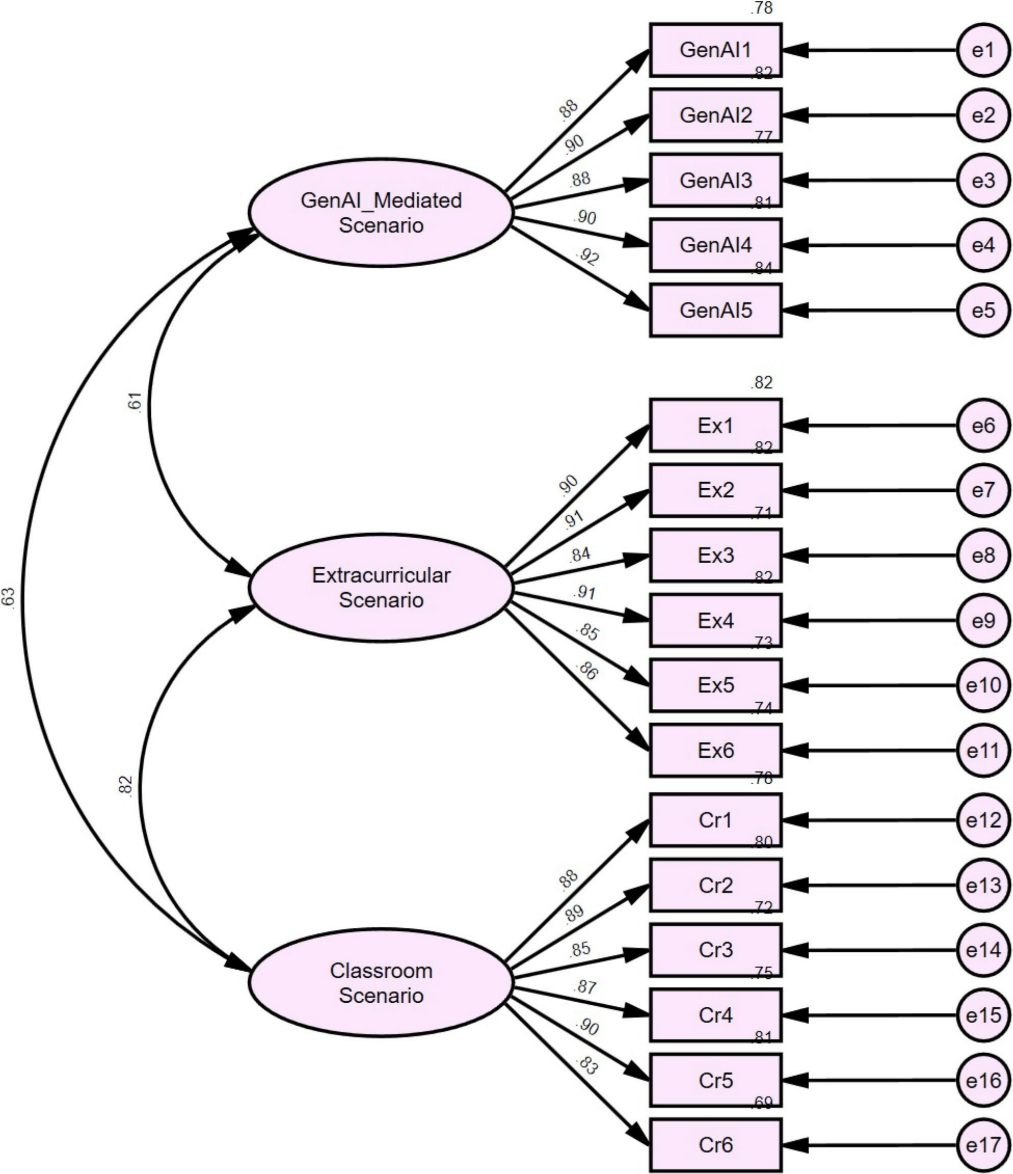
Model of the Multi-scenario EFL WTC Scale

To evaluate whether the scale met acceptable model fit criteria, confirmatory factor analysis (CFA) was conducted using the second subsample. As shown in Table 2; Fig. 1, the overall model demonstrated good fit. Specifically, χ²/df = 4.975 was slightly higher than the commonly recommended threshold (< 3); however, given the large sample size, this value remains within an acceptable range. The RMSEA (0.079) and SRMR (0.032) were both below their respective recommended cutoffs of 0.08 and 0.05. Additionally, both the CFI (0.961) and TLI (0.954) exceeded the conventional benchmark of 0.90. In summary, the measurement model exhibits adequate fit to the observed data and reasonably represents the relationships between the latent constructs and their corresponding observed indicators.

**Table 2:** Model Fit Results

	χ2	df	χ2/df	RMSEA	SRMR	TLI	CFI
Model fit	577.065	116	4.975	0.079	0.032	0.954	0.961
Recommended cutoff value			< 3	< 0.08	< 0.05	> 0.9	> 0.9

χ2  Chi-square, *df*  degrees of freedom

The study examined convergent validity using standardized factor loadings, Composite Reliability (CR), and Average Variance Extracted (AVE). Results in Table 3 showed that all standardized factor loadings ranged from 0.832 to 0.919 (*p* < 0.001), exceeding the recommended threshold of 0.70 [35]. Furthermore, the CR values for all constructs were above 0.90, and the AVE values all exceeded 0.75—substantially higher than the conventional benchmarks of CR ≥ 0.70 and AVE ≥ 0.50 [36] (Hair et al. 2010). These results indicate strong associations between the observed items and their respective latent constructs, confirming excellent convergent validity.

**Table 3:** Convergent Validity Results

Component	Item	UnSd.E.	Sd.E.	S.E.	C.*R*.	*p*	CR	AVE
GenAi-mediated scenario	GenAI1	1	0.882				0.953	0.804
	GenAI2	1.046	0.903	0.031	33.878	***		
	GenAI3	0.997	0.878	0.031	32.089	***		
	GenAI4	1.018	0.900	0.030	33.433	***		
	GenAI5	1.051	0.919	0.030	35.065	***		
Extracurricular scenario	Ex1	1	0.904				0.953	0.772
	Ex2	0.991	0.905	0.027	37.036	***		
	Ex3	0.885	0.843	0.029	30.578	***		
	Ex4	0.962	0.906	0.027	36.251	***		
	Ex5	0.944	0.855	0.030	31.493	***		
	Ex6	0.877	0.859	0.028	31.723	***		
Classroom scenario	Cr1	1	0.883				0.949	0.757
	Cr2	1.028	0.892	0.031	33.263	***		
	Cr3	0.906	0.847	0.031	29.672	***		
	Cr4	0.967	0.866	0.031	30.828	***		
	Cr5	1.026	0.898	0.031	33.531	***		
	Cr6	0.956	0.832	0.034	28.442	***		

This study employed the Fornell–Larcker criterion to assess discriminant validity. Specifically, the square root of the Average Variance Extracted (AVE) for each latent construct was compared with its correlations with all other latent constructs. According to Fornell and Larcker Criterion [36], discriminant validity is established when the square root of a construct’s AVE is greater than its correlations with any other construct. As shown in Table 4, the square roots of the AVE values on the diagonal (0.896, 0.879, and 0.870) are all larger than the corresponding inter-construct correlations in their respective rows and columns. This indicates that each latent construct shares more variance with its own items than with items of other constructs, demonstrating strong independence among the three components and confirming that the measurement model possesses good discriminant validity.

**Table 4:** Discriminant Validity Results

Component	1	2	3
1. GenAi-mediated scenario	**0.896**		
2. Extracurricular scenario	0.611***	**0.879**	
3. Classroom scenario	0.627***	0.819***	**0.870**

Diagonal values (in bold) represent the square roots of the AVE for each construct. Off-diagonal values are Pearson correlations between latent constructs

*** Indicates *p*< 0.001

### Criterion-related validity

To examine criterion-related validity, the Scale of WTC in English was used as the criterion measure. Correlation analyses were conducted between the mean scores of each subscale and the total score of the newly developed scale and the criterion scale. As shown in Table 5, all subscales and the total score of the new scale were significantly correlated with the Scale of WTC in English, with correlation coefficients ranging from 0.649 to 0.874 (*p* < 0.001). These results indicate that the new scale demonstrates strong external criterion-related validity.

**Table 5:** Criterion-Related Validity Results

	1	2	3	4	5
1. Scale of WTC in English	—				
2. GenAI-mediated scenario	0.649***	—			
3. Extracurricular scenario	0.796***	0.591***	—		
4. Classroom scenario	0.848***	0.600***	0.786***	—	
5. Multi-scenario EFL WTC Scale	0.874***	0.807***	0.915***	0.913***	—

*** indicates* p*< 0.001

### Multi-group confirmatory factor analysis

To examine whether the scale demonstrates measurement invariance across gender and household registration status, multi-group confirmatory factor analysis (MG-CFA) was conducted using the third subsample, with gender (male vs. female) and household registration (urban vs. rural) as grouping variables. Four nested models were tested sequentially: configural invariance, metric invariance, scalar invariance, and strict invariance. As shown in Table 6, the changes in model fit indices across increasingly constrained models met established invariance criteria: ΔCFI and ΔTLI were all below 0.01 [33] and ΔRMSEA was less than 0.015 [34]. These results indicate that the scale achieves full measurement invariance across both gender and household registration groups. Consequently, the factor structure of the scale is stable across these subgroups, and the scores are comparable across populations.

**Table 6:** MG-CFA Results

Group	Model	χ2	df	χ2/df	Δχ2	Δdf	*p*	CFI	ΔCFI	TLI	ΔTLI	RMSEA	ΔRMSEA
household registration	Unconstrained	1004.463	232.000	4.330	—	—	—	0.944	—	0.935	—	0.073	—
	Measurement weights-Configural invariance	1015.253	246.000	4.127	10.790	14.000	0.703	0.945	0.001	0.939	0.004	0.070	-0.003
	Measurement intercepts-Metric invariance	1033.026	263.000	3.928	17.773	17.000	0.403	0.945	0.000	0.943	0.004	0.068	-0.002
	Structural covariances-Scalar invariance	1068.644	269.000	3.973	35.618	6.000	< 0.001	0.942	-0.003	0.942	-0.001	0.069	0.001
	Measurement residuals-Residual Invariance	1137.556	286.000	3.977	68.912	17.000	< 0.001	0.939	-0.003	0.942	0.000	0.069	0.000
Gender	Unconstrained	1050.768	232.000	4.529	—	—	—	0.941	—	0.931	—	0.075	—
	Measurement weights-Configural invariance	1070.104	246.000	4.350	19.336	14.000	0.153	0.940	-0.001	0.934	0.003	0.073	-0.002
	Measurement intercepts-Metric invariance	1100.552	263.000	4.185	30.448	17.000	0.023	0.939	-0.001	0.937	0.003	0.071	-0.002
	Structural covariances-Scalar invariance	1131.973	269.000	4.208	31.421	6.000	< 0.001	0.938	-0.001	0.937	0.000	0.071	0.000
	Measurement residuals-Residual Invariance	1231.357	286.000	4.305	99.384	17.000	< 0.001	0.932	-0.006	0.935	-0.002	0.072	0.001

## Discussion

This study aimed to develop and validate a Multi-scenario EFL WTC Scale to assess learners’ willingness to use English across diverse contexts—specifically, classroom, extracurricular, and GenAI-mediated settings. The findings indicate that the scale demonstrates strong psychometric properties, including clear factor structure, satisfactory reliability, and solid construct validity. In addition, the results of multi-group confirmatory factor analysis support full measurement invariance across gender and household registration (urban vs. rural), suggesting that the scale functions consistently across these demographic groups. Overall, the scale provides a reliable and valid instrument for examining learners’ willingness to communicate in an increasingly diverse communicative landscape that now includes both traditional interpersonal interaction and emerging human–AI communication. However, these findings should be interpreted with some caution, as the sample in the present study was relatively restricted, which may limit the generalizability of the results to broader populations of EFL learners.

The Multi-scenario EFL WTC Scale developed in this study encompasses a range of English communication scenarios across three contexts: classroom, extracurricular, and GenAI-mediated scenarios. First, with respect to the classroom and extracurricular dimensions, our findings align with prior research—specifically, studies that examine learners’ willingness to use language in formal instructional settings [16] and informal, everyday contexts [17]. The key distinction of the present study lies in its incorporation of GenAI tools as an emerging communicative medium. Unlike traditional WTC research that emphasizes interpersonal interaction, this study focuses on learners’ willingness to engage in human–AI interaction, particularly their tendency for language output and proactive engagement when communicating with AI systems. This extension reflects current trends in technology-enhanced language learning and opens new avenues for conceptualizing the scope of WTC.

The inclusion of the GenAI-mediated environment as a core dimension of WTC is primarily grounded in its demonstration of quasi-interpersonal interaction characteristics across three critical aspects. First, in terms of interaction, GenAI systems demonstrate human-like conversational capabilities, including context-aware multi-turn dialogue, real-time feedback mechanisms that mimic natural turn-taking, and intelligent modules capable of recognizing learners’ emotional states and generating empathetic responses. This highly realistic interaction pattern creates a language environment closely resembling authentic interpersonal communication. Second, at the linguistic level, current GenAI tools can automatically adjust lexical difficulty and syntactic complexity according to the learner’s proficiency. This personalized linguistic support ensures that the communicative content remains both challenging and comprehensible. Thirdly, regarding the motivation to communicate, GenAI tools effectively reduce learners’ anxiety and enhance their communicative confidence through motivational strategies such as encouraging feedback and acknowledgment of progress. This affective support mechanism produces psychological effects similar to those provided by human teachers. Therefore, incorporating the GenAI-mediated environment as an independent dimension in modern WTC measurement instruments represents not only a necessary extension of traditional theories of WTC in English, but also a responsive adaptation to the evolving digital language learning ecosystem. This approach enables a more comprehensive and accurate assessment of learners’ WTC across diverse linguistic contexts.

The GenAI-mediated WTC scenario represents a distinct construct when compared to existing models of WTC in digital contexts (e.g., online peer interaction). While digital contexts typically refer to communication with human interlocutors via digital platforms, where the interaction remains fundamentally human despite the mediation of technology, GenAI-mediated WTC involves interaction with artificial intelligence systems. A key distinction of GenAI-mediated WTC lies in the AI’s ability to provide personalized, context-sensitive feedback and its capacity to engage in non-judgmental interactions. This non-evaluative environment fosters a unique, pressure-free space for language practice, thereby facilitating learner autonomy and confidence in a manner that differs from traditional online peer interactions. Consequently, the unique features of GenAI-mediated WTC, such as the AI’s individualized responses and the absence of human judgment, justify its classification as an independent dimension within the broader framework of WTC constructs.

Second, regarding the content of the scale items, all items—whether in the classroom, extracurricular, or GenAI-mediated scenarios—are centered on the core construct of “willingness to use English for communication.” The only variation lies in the situational context and specific form of the communicative activity, which faithfully reflects the essential nature of WTC. The item pool encompasses a wide range of communicative tasks, such as expressing opinions, seeking help, role-playing, and participating in speech contests, illustrating that WTC is not merely an attitudinal disposition but also manifests as a tendency toward concrete language behaviors.

Finally, the results of the confirmatory factor analysis revealed a high covariance coefficient (0.820) between classroom and extracurricular WTC, indicating a strong association between these two latent constructs. This substantial overlap suggests that, in learners’ perceptual frameworks, classroom and extracurricular contexts may not be sharply differentiated as distinct communicative arenas. Instead, they appear to share common social and affective foundations—such as interaction with human interlocutors, the possibility of evaluation, and opportunities for immediate social feedback—which together contribute to a unified sense of interpersonal willingness to communicate in English. From an ecological perspective [37], WTC is embedded within multiple interconnected systems (e.g., the microsystem of the classroom and the mesosystem linking classroom and extracurricular contexts), where different contexts are interconnected through shared social–affective processes.

Unlike the two human-centered dimensions, interaction with AI systems introduces unique features—including non-judgmental feedback, personalized adaptation without social evaluation, reduced interpersonal anxiety, and a pressure-free practice space—that fundamentally alter the psychological dynamics of WTC. This distinction is further evidenced by the lower covariance and higher discriminant validity between the GenAI dimension and the other two, highlighting how emerging human–AI communication creates a novel layer of willingness that extends beyond traditional interpersonal boundaries. These findings thus reinforce the theoretical and empirical justification for maintaining three separate yet interrelated dimensions in the multi-scenario WTC model: while classroom and extracurricular WTC reflect overlapping facets of social-interpersonal communication, GenAI-mediated WTC captures an emerging, technology-facilitated domain with distinct motivational and affective characteristics.

The results of the multi-group invariance analysis provide important evidence for the robustness of the scale across different subgroups. The established invariance across gender and household registration suggests that the scale measures WTC in a consistent manner across these groups, allowing for meaningful comparisons without measurement bias. This finding also implies that the underlying psychological mechanisms of EFL learners’ willingness to communicate may be relatively stable across these demographic characteristics. From a practical perspective, this enhances the applicability of the scale in diverse educational contexts and supports its use in comparative studies involving different learner populations.

## Implications

This study aimed to develop and validate a Multi-scenario EFL WTC Scale to assess learners’ willingness to use English across diverse contexts—classroom, extracurricular, and GenAI-mediated scenarios. The research holds significant theoretical and practical value. Theoretically, by constructing a multidimensional WTC scale that integrates classroom, extracurricular, and GenAI-mediated scenarios, this study moves beyond the traditional conceptualization of WTC, which has been largely confined to human-to-human interaction. It pioneers the inclusion of the GenAI environment as an independent dimension in WTC measurement, offering a novel theoretical lens for understanding the psychological mechanisms of language learning in intelligent technological settings. This perspective also aligns with dialogic views of language and learning. For example, Bakhtin’s dialogism posits that language is fundamentally dialogic [38], as every utterance responds to previous voices while anticipating future responses, and meaning emerges through this ongoing interaction. In this regard, the proposed scale provides a useful instrument for examining whether and how different interactional contexts—particularly those involving GenAI—encourage learners to participate in dialogue and increase their willingness to communicate in English. Practically, the validated scale enables educators to accurately evaluate learners’ WTC profiles across different communicative contexts. This insight supports the design of differentiated instructional strategies—optimizing both conventional classroom and informal learning activities while also providing evidence-based guidance for the pedagogical integration of GenAI tools in language education. From a dialogic perspective, the findings also highlight how different communicative environments—including human–human and human–AI interactions—may shape learners’ willingness to engage in meaningful language exchange. This understanding can help educators create more supportive and interactive learning environments that encourage active participation and sustained dialogue. Ultimately, the scale contributes to enhancing learners’ communicative competence and digital literacy, playing a meaningful role in advancing innovative, technology-informed language teaching in the era of artificial intelligence.

## Limitations and suggestions for future research

Although this study has made certain progress, several limitations remain and should be addressed in future research. First, test-retest reliability was not examined, and therefore, the stability and consistency of the scale over time cannot be assessed. To enhance the reliability evidence of the scale, future studies could design longitudinal research and collect multiple measurements to validate the scale’s stability at different time points. In particular, longitudinal designs would allow researchers to examine both the stability and developmental trajectories of WTC across time, thereby providing stronger evidence for the temporal robustness of the scale. Additionally, comparing results from different time intervals could assess the test-retest reliability, thereby better examining the effectiveness of the scale for long-term use.

Second, the current sample was limited to university students, which restricts the generalizability of the findings. The characteristics of the university student group may differ from those of other populations, and as such, the scale may not adequately capture the WTC characteristics of learners with different ages, educational backgrounds, or proficiency levels. Therefore, future research should expand the sample to include secondary school students, beginner learners, and adult English learners, as well as individuals from diverse cultural and national backgrounds to improve the external validity and applicability of the results. Such extensions would enable more robust cross-group validation of the scale and help determine whether the factor structure and measurement properties remain stable across different populations. By including these more diverse samples, the research will be able to more comprehensively assess the scale’s applicability and variability across different groups.

Third, despite the relatively large overall sample size and the use of multiple subsamples for scale development and validation, the gender distribution was imbalanced, with female participants substantially outnumbering male participants. Although measurement invariance across gender was supported through MG-CFA, future research should replicate the validation process using more gender-balanced samples to further strengthen the generalizability of the findings.

Finally, the scale development and validation in this study were primarily based on Classical Test Theory. Although Classical Test Theory is widely used in psychometrics, it is sample-dependent and offers limited item-level insights. Therefore, future research could incorporate Item Response Theory to complement Classical Test Theory. Item Response Theory provides more detailed evaluations of item difficulty, discrimination, and guessing parameters, helping researchers understand how each item performs across different proficiency levels. In this way, IRT can address one major limitation of CTT by providing sample-independent parameter estimation and more precise information about how individual items function across different levels of the latent trait. Furthermore, network analysis can serve as a supplementary method for cross-validating the scale’s dimensional structure. It can also reveal relationships between items. Unlike CTT, which primarily focuses on total scores and latent factor structures, network analysis can illuminate the direct connections among items (or *nodes* in network analysis) and identify which nodes are most central within the WTC construct. This approach can therefore offer a more dynamic understanding of the internal organization of the scale and reveal patterns that may not be captured through CTT alone. Combining Item Response Theory with network analysis will allow for a deeper understanding of the scale’s dynamics and further enhance its validity and interpretability. This integration will strengthen the scale’s theoretical depth and practical applicability, supporting its continued optimization.

## Conclusion

This study reconceptualizes WTC in the AI era by validating GenAI-mediated interaction as a distinct dimension alongside classroom and extracurricular scenarios. The resulting scale provides researchers and educators with a useful tool to examine and support learners’ communication willingness across diverse scenarios. Ultimately, by bridging the gap between theoretical models and the realities of AI-integrated learning, this research paves the way for a more adaptive and inclusive approach to fostering global communicative competence.

## Supplementary Information

Supplementary Material 1.

## Data Availability

The datasets generated and/or analyzed during the current study are available from the corresponding author upon reasonable request for non-commercial research purposes.
