# Supplementary material for: Developing and validating multi-scenario EFL willingness to communicate scale across classroom, extracurricular, and GenAI-mediated scenarios
Source: BMC Psychol. 2026 Apr 22;14:819. doi: 10.1186/s40359-026-04607-1 (PMC13234994; doi:10.1186/s40359-026-04607-1)
Supplement: Supplementary file 1 — Supplementary Material 1. [file 40359_2026_4607_MOESM1_ESM.docx]

Multi-scenario EFL Willingness to Communicate Scale across Classroom, Extracurricular, and GenAI-mediated Contexts

GenAI-mediated Scenario

[1] I am willing to use English to simulate everyday conversations with generative AI tools (such as Doubao).

[2] I am willing to use English to simulate interview situations with generative AI tools (such as Doubao).

[3] I am willing to use English to discuss or debate viewpoints with generative AI tools (such as exploring social issues, technological developments, etc.).

[4] I am willing to use English to seek help from generative AI tools (such as improving my English pronunciation).

[5] I am willing to use English to engage in business or academic conversations with generative AI tools (such as Doubao) in order to improve my formal communication skills.

Classroom Scenario

[1] In English class, I am willing to answer the teacher’s questions in English.

[2] In English class, I am willing to communicate with my classmates in English.

[3] In English class, I am willing to read the text aloud in English.

[4] In English class, I am willing to complete tasks assigned by the teacher in English, such as role-playing.

[5] In English class, I am willing to introduce myself in English.

[6] In English class, I am willing to ask my classmates questions in English about things I do not understand.

Extracurricular Scenario

[1] Outside of class, I am willing to participate in English debate competitions.

[2] Outside of class, I am willing to participate in English speech contests and interact with judges.

[3] Outside of class, I am willing to use social media or communication tools to chat in English with people who have English proficiency.

[4] Outside of class, I am willing to join English-related clubs or activities, such as English corners or speaking clubs.

[5] Outside of class, I am willing to chat with my classmates in English.

[6] Outside of class, I am willing to communicate in English with foreigners I encounter.
